# The Effects of Lack of Awareness in Age-Related Quality of Life, Coping with Stress, and Depression among Patients with Malignant Melanoma

**DOI:** 10.3390/curroncol30020116

**Published:** 2023-01-23

**Authors:** Ana-Olivia Toma, Estera Boeriu, Luminita Decean, Vlad Bloanca, Felix Bratosin, Mihaela Codrina Levai, Neeharika Gayatri Vasamsetti, Satish Alambaram, Andrada Licinia Oprisoni, Bogdan Miutescu, Kakarla Hemaswini, Iulius Juganaru, Andrei-Cristian Bondar, Marius Liviu Moise

**Affiliations:** 1 Department of Microbiology, “Victor Babes” University of Medicine and Pharmacy, Eftimie Murgu Square 2, 300041 Timisoara, Romania; 2 Department of Pediatrics, Discipline of Pediatric Oncology and Hematology, “Victor Babes” University of Medicine and Pharmacy Timisoara, Eftimie Murgu Square 2, 300041 Timisoara, Romania; 3 Faculty of General Medicine, George Emil Palade University of Medicine, Pharmacy, Science and Technology, Strada Gheorghe Marinescu 38, 540139 Targu Mures, Romania; 4 Department of Plastic Surgery, “Victor Babes” University of Medicine and Pharmacy Timisoara, Eftimie Murgu Square 2, 300041 Timisoara, Romania; 5 Methodological and Infectious Diseases Research Center, Department of Infectious Diseases, “Victor Babes” University of Medicine and Pharmacy, 300041 Timisoara, Romania; 6 Research Center for Medical Communication, “Victor Babes” University of Medicine and Pharmacy, 300041 Timisoara, Romania; 7 Faculty of General Medicine Nizampura, Kaloji Narayana Rao University of Health Sciences, Warangal 506007, India; 8 Bhaskar Medical College, Amdapur Road 156-162, Hyderabad 500075, India; 9 Department of Gastroenterology and Hepatology, “Victor Babes” University of Medicine and Pharmacy Timisoara, Eftimie Murgu Square 2, 300041 Timisoara, Romania; 10 Malla Reddy Institute of Medical Sciences, Suraram Main Road 138, Hyderabad 500055, India; 11 Department of Pediatrics, “Victor Babes” University of Medicine and Pharmacy, Eftimie Murgu Sq. No. 2, 300041 Timisoara, Romania; 12 Psychiatry Hospital “Prof. Dr. Alexandru Obregia”, Soseaua Berceni 10, 041914 Bucuresti, Romania; 13 Department of Radiology, “Premiere” Hospital—“Regina Maria”, Calea Aradului 113, 300643 Timisoara, Romania

**Keywords:** skin cancer awareness, malignant melanoma, quality of life, coping with stress

## Abstract

Almost one-third of all malignant melanoma patients exhibit emotional stress indicating the need for professional care. Considering this, patients’ psychological needs are routinely overlooked and unfulfilled, even though there is substantial evidence that psychological therapies may enhance psychosocial outcomes for melanoma patients, such as low mood, sadness, and anxiety. Among developing countries and some health systems in developed regions, the lack of awareness and screening methods for skin cancer creates a high risk of psychological issues associated with more advanced diseases. Therefore, the current study aimed to investigate and compare the impact of malignant melanoma awareness for screening, prevention, and treatment on the patient’s quality of life and coping with stress and depression, based on patients’ age. This cross-sectional study recruited 238 patients with malignant melanoma distributed into two groups, Group A patients between 18 and 65 years and Group B patients older than 65. There were no significant gender differences and cancer staging differences between groups, although self-reported depressed mood and anhedonia were significantly more frequent in younger adults with malignant melanoma (43.8% vs. 28.9%). From the unstandardized surveys, it was observed that significantly fewer patients from Group B knew that melanoma could be caused by sun exposure (34.2% vs. 52.2%), and they were less likely to use sunscreen or visit a doctor to evaluate their skin moles (25.9% vs. 14.5%). Elderly patients preferred television as the main source of information, and only 68.4% of patients from Group B were using smart devices. There was a significantly higher physical score on the SF-12 scale among Group A patients, although patients from Group B scored higher in the mental health assessment, and the perceived helplessness on the PSS-10 scale was significantly higher compared to younger adults with melanoma (2.97 vs. 2.71, *p*-value = 0.036). Lower scores on the physical and mental SF-12 questionnaire determined a higher presence of depressive symptoms (rho = −0.352, respectively rho = −0.273). Higher scores on the DLQI sexual difficulties and treatment difficulties also correlated significantly with the presence of depressive symptoms and anhedonia (rho = 0.341, respectively rho = 0.264). Awareness campaigns for malignant melanoma should focus on the elderly population, too, using the television as the main communication channel. On the other hand, the more informed and knowledgeable group of adults younger than 65 are more likely to experience psychological problems and should be targeted for psycho-oncological aid.

## 1. Introduction

Malignant melanoma prevalence seems to rise globally [1,2]. The incidence of melanoma continues to rise in both adult and young populations. Although melanoma is uncommon in children, the chance of acquiring it greatly increases in teenagers and young adults, when it is the second most prevalent form of cancer [3]. Relative to adult patients, the incidence of melanoma decreased significantly among those aged 15 to 29 (by 4% to 6% yearly) but remained consistent among those older than 30 [4]. Research into malignant melanoma and treatments for the affected patients have also increased [5,6]. The introduction of immunotherapies into clinical practice has greatly improved the prognosis and outlook for people with malignant melanoma. In recent years, significant progress has been made in scientific and clinical development, with breakthrough knowledge and treatment of melanoma currently being extended to other types of cancer [7,8]. Unfortunately, incidence, recurrence, and mortality rates continue to be alarmingly elevated, and considerable work is needed to resolve basic problems about melanoma control, screening, identification, and management [9].

Due to the growing prevalence of skin cancer and detection technologies, as well as the authorization of novel treatment modalities for advanced skin cancer, there is a growing necessity for patient awareness and information [10,11]. For instance, patients wish they had a better understanding of how to prevent skin cancer, how to diagnose it, and the possible side effects of immunotherapy [12]. This was indicated by the current information-seeking behavior of melanoma patients, whose utilization of contemporary media-accessible resources has significantly changed [13].

Along with the rise in the prevalence of skin cancer, there has been an increase in focus on the psychological impact of malignant melanoma on patients’ lives [14,15]. In addition, in response to the growing incidence and demand for skin cancer-related education, it is hypothesized that a greater awareness of the methods available to prevent, diagnose, and treat an aggressive malignancy, such as malignant melanoma, can improve the patient’s quality of life, facilitate disease stress management, and prevent depression.

The analysis of individual perspectives indicates difficulties after oncological treatment, conceptualized as serious life disturbances and identity issues. Although the individual was no longer sick, a persistent worry of recurrence and physical changes prohibited a return to the typical pre-cancer condition in terms of health, identity, and relationships [16,17]. Since oncological therapies cause scarring, weight gain, and other adverse effects, the interaction with the body is an important topic. To enhance general well-being following illness, individualized psychological care addressing physical symptoms and associated emotions and thoughts should be offered [18].

Regarding sexuality, body image, and relationship consequences, additional research have shown that breast cancer patients have considerably higher rates of sexual dysfunction and lower body image compared to healthy women. Despite this fact, the majority of breast cancer patients are not satisfied with the quantity and quality of sexuality-related treatment they get from their healthcare professionals [19]. However, these studies primarily examine female patients, despite the fact that there are significant variations between male and female populations. A comparable problem, the diagnosis of malignant melanoma might have different repercussions than the diagnosis of breast cancer due to the greater death rate in the former situation. At the same level of concern, cancer survivors are at a higher risk of developing decreased body acceptance and intimacy problems, and have a weaker correlation than the healthy control group between manageability and meaningfulness and an appropriate attitude toward food and intimate relationship, respectively. A greater display of femininity in the treated group may be seen as a beneficial, socio-culturally conditioned coping technique [20]. Despite this, social support becomes crucial for cancer patients, given the aforementioned concerns that impact their quality of life. Nevertheless, not all forms of assistance are successful, since patients’ opinions and participation requirements in the healthcare process vary. Future experimental studies might investigate the association between social support, personal needs, and personality traits in more detail [21].

Therefore, the current study aimed to investigate the impact of malignant melanoma awareness for screening, prevention, and treatment on the patient’s quality of life and coping with stress and depression based on the patients’ age. The primary objective was to determine if there is a difference in the coping strategies, quality of life, and stress levels between younger adults and elderly patients with a diagnosis of malignant melanoma, while the secondary objective was to analyze their demographics and evaluate the level of information, misinformation, and disease awareness among younger adults and elderly patients with malignant melanoma.

## 2. Materials and Methods

### 2.1. Study Design and Ethics

The current research was designed as a cross-sectional study using patient-filled questionnaires. The duration of the study was one year, from October 2021 to October 2022. The study was organized as a multi-centric research involving the Dermatology unit and the Plastic Surgery Department of the Victor Babes University of Medicine and Pharmacy from Timisoara, Romania. The current study strictly followed the guidelines and regulations of the Local Commission of Ethics for Scientific Research, operating under the EU GCP directives and with the Declaration of Helsinki—Recommendations Guiding Medical Doctors in Biomedical Research Involving Human Subjects. The participating patients were not paid or given additional benefits compared to the other patients of these two clinics.

### 2.2. Patient Selection Process and Surveying Methods

Patients attending these two clinics were identified from the electronic database by a confirmed diagnosis of malignant melanoma according to the ICD-10 standard of diagnosing diseases [22]. All patients were presented with the questionnaires and asked during their hospital visits if they were willing to participate in a study without knowing the aim of the study, to avoid biased results. Two researchers were involved in the distribution of the online questionnaires to the patients who consented to participate in the study and have their medical records evaluated in addition to the surveys. Instructions were provided on how to fill the surveys. Patients who did not have a digital support to fill the questionnaires were asked to fill in the printed questionnaires. Another group of two researchers who participated in this study were given the task of collecting the filled questionnaires and gathering data in a Microsoft Excel spreadsheet. Patients were split into two groups by their age: Group A—the adult patients from 18 to 65 years, and Group B—the elderly patients from the age of 65 years. The two patient groups were considered to be split by age difference since the age of 65 is considered as the retirement age in Romania, while studies show that the incidence of melanoma is significantly increased in patients older than 65 years; elderly patients with cancer also exhibit different stress levels and emotional distress [23,24,25,26]. The sample size was calculated using a confidence level of 95% and a margin of error of 5%, based on the prevalence of malignant melanoma in the general Caucasian population of approximately 2.5% [27].

The study comprised a part of an unstandardized questionnaire with questions designed by the research team, as well as a standardized part using recognized and validated questionnaires that were translated into the native language of the participants (Romanian). The following questionnaires were used: (1) Coping Orientation to Problems Experienced Inventory (COPE-16), with a Cronbach alpha value of 0.70 [28,29]; (2) 12-Item Short Form Survey (SF-12), with a Cronbach alpha value between 0.72 and 0.89 [30,31]; (3) the Dermatology Life Quality Index (DLQI) (Cronbach’s alpha = 0.83) [32]; and (4) the Perceived Stress Scale (PSS-10), with a Cronbach alpha range between 0.81 and 0.88 [33].

### 2.3. Data Collection and Variables

The inclusion criteria in the current study were the following: (1) patients being 18 years or older; (2) patients having a confirmed diagnosis of malignant melanoma; (3) patients having the ability to consent and willingness to participate in the study; (4) all questionnaires should be correctly and completely filled by the participants. Patients with incomplete questionnaires or medical records were excluded from the study, as well as those who were underage, lacking consent, or having mental impairments. Further, patients with stage IV cancer, according to the American Joint Committee on Cancer (AJCC) TNM staging system, were not considered for inclusion [34]. Another exclusion criterion was ongoing chemotherapy, immunotherapy, or radiotherapy, to avoid biased results. Two researchers were involved in data collection during the one-year period of the study, gathering data from the filled surveys into a standardized MS Excel worksheet. The variables considered for inclusion in the current study comprised the following: (1)—patients’ demographics (age group, gender, area of residence, relationship status, number of comorbidities, substance use behavior, history of major depression or current depressed mood or anhedonia, malignant melanoma staging); (2)—the unstandardized surveys with questions related to awareness and misinformation for malignant melanoma prevention, screening, and treatment; and (3)—the standardized questionnaires (COPE-60, SF-12, PSS-10, and the DLQI survey).

### 2.4. Statistical Analysis

Data were obtained electronically and de-identified. Mean values and standard deviations (SD), *p*-values, and correlation coefficient “r” of the values were calculated using the statistical analysis software MedCalc v.20 (MedCalc Software bv, Ostend, Belgium). Variables were compared between group A and group B, including the above-mentioned variables. The Student’s t-test was used to compare variables described by means and standard deviation. Chi-square and Fischer’s exact tests were applied to verify a possible difference between the two groups regarding variables described as proportionate values. Pearson and Spearman’s correlation analysis methods were used to calculate the “r” coefficient of correlation. A *p*-value < 0.05 was considered statistically significant when comparing the study variables.

## 3. Results

### 3.1. Demographical Data

During the study period, a total of 287 patients with a confirmed diagnosis of malignant melanoma were asked to participate in this study. Out of 261 who consented to participate and complete the surveys, a total of 238 questionnaires were returned correctly and completely filled. Among the younger adult population (Group A), there were a total of 162 patients, while 76 older patients were included in Group B, as seen in Table 1. There were no gender differences between the two study groups, with 45.7% women in Group A, and 47.4% in Group B. It was observed that the younger adult patients with malignant melanoma in Group A had a mean age at diagnosis of 43.0 years, while the elderly patients in Group B had a mean age of diagnosis of 67.2 years (t = 14.315, SE = 1.69, df = 236, *p*-value < 0.001). There was a significantly higher proportion of elderly patients residing in the urban regions (69.7% vs. 56.2%, *p*-value = 0.045). Frequent alcohol consumption was also significantly more common in Group B (22.4% vs. 12.3%, *p*-value = 0.046).

In correlation with the age difference between the two study groups, the number of comorbidities was significantly higher in Group B of patients older than 65 years, 35.5% of them having three or more comorbid conditions, compared to Group A with 15.4% having three or more comorbidities (*p*-value < 0.001). Lastly, it was observed that younger patients with malignant melanoma had a more frequent history of major depression (11.7% vs. 7.9%), although the difference was not statistically significant. However, self-reported depressed mood and anhedonia were significantly more frequent in younger adults with malignant melanoma (43.8% vs. 28.9%, *p*-value = 0.028). Regarding the cancer staging of the studied patients, there was no significant difference (63.6% stage III in Group A vs. 50.0% stage III in Group B).

### 3.2. Unstandardized Survey

The unstandardized survey comprised a total of 11 questions aiming to determine the level of information, misinformation, and awareness about the screening, prevention, and treatment of malignant melanoma. It was observed that significantly fewer patients from Group B knew that melanoma could be caused by sun exposure (34.2% vs. 52.2%). Only 68.4% of the elderly patients were using a smartphone, tablet, or personal computer, compared to 90.7% in Group A (*p*-value < 0.001). In addition, the elderly patients were more likely to prefer television as a source of information, compared to younger adults from Group A, who preferred the internet as the main source of information, as seen in Table 2. The elderly patients from Group B with malignant melanoma were significantly less likely to use sunscreen and visit a doctor to evaluate the skin moles (25.9% vs. 14.5%, *p*-value = 0.047), respectively (23.5% vs. 9.2%, *p*-value = 0.009). Lastly, the majority of patients believed that their condition could have been prevented if they had known better about the prevention and screening of malignant melanoma (66.7% in Group A vs. 80.3% in Group B, *p*-value = 0.031).

### 3.3. Standardized Surveys

**Figure 1:**
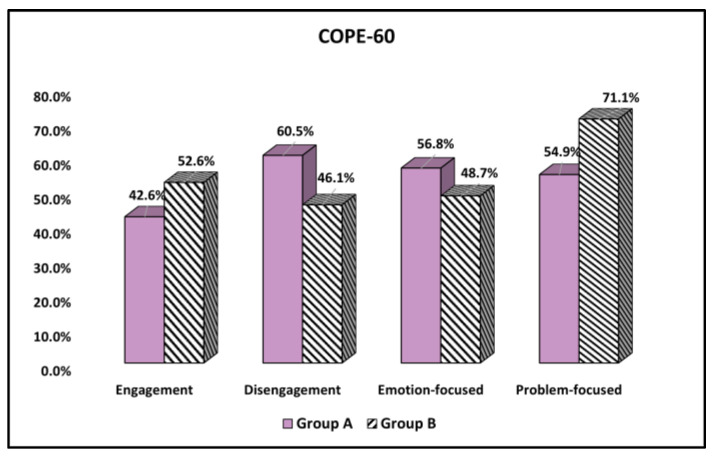
Comparison of COPE-60 questionnaire results by age group.

The analysis of the COPE-60 results is described in Table 3 and Figure 1, with stratification of results by patients’ age. It was observed that a significantly higher proportion of younger adult patients with malignant melanoma from Group A were using disengagement methods for coping with the stress of their disease compared with the elderly patients (60.5% vs. 46.1%, *p*-value = 0.036). In contrast, the elderly patients were significantly more likely to use problem-focused coping methods (71.1% vs. 54.9%, *p*-value = 0.018).

**Figure 2:**
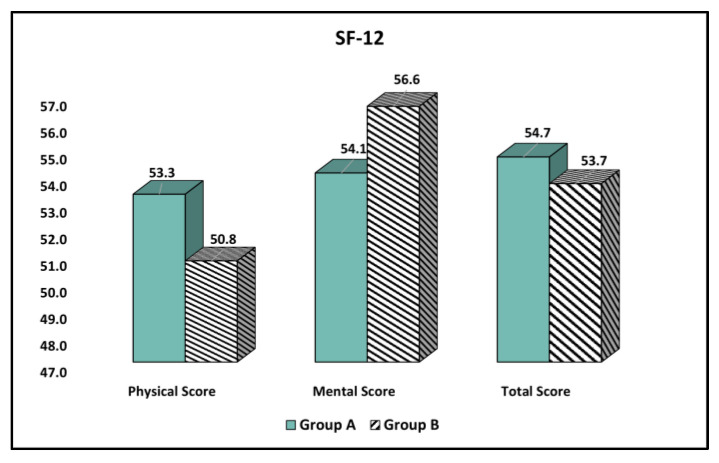
Comparison of quality of life physical and mental scores based on the SF-12 questionnaire stratified by age group.

The comparison of quality of life physical and mental scores based on the SF-12 questionnaire stratified by age group is presented in Table 4. There was a significantly higher physical score among Group A patients (55.2 vs. 50.8, *p*-value < 0.001), although the elderly patients with malignant melanoma from Group B scored significantly higher in the mental health assessment, with a score of 56.6 compared to 54.1 in Group A (*p*-value = 0.026). However, the total score was not significantly different between the groups, as seen in Figure 2.

Questions presented in Table 5 describe ten items based on the DLQI questionnaire that ask the patients how much they consider their skin cancer diagnosis is causing each of the presented issues. It was observed that a significantly higher proportion of young adults from Group A experienced embarrassment compared to Group B patients (16.0% vs. 6.6%, *p*-value = 0.043). On the other hand, the elderly patients with melanoma from Group B believed their condition was creating sexual difficulties (46.1% vs. 25.2% in Group A, *p*-value = 0.002). Group B patients also described treatment difficulties in a significantly higher proportion (67.1% vs. 29.6%, *p*-value < 0.001).

**Figure 3:**
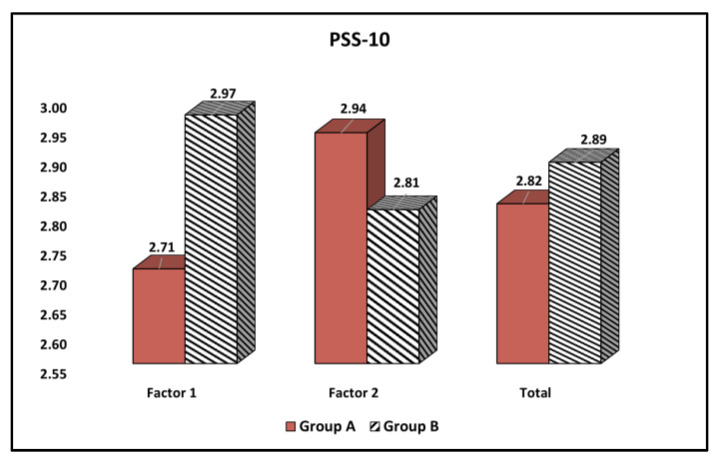
Comparison of quality of stress levels based on the Perceived Stress Scale (PSS-10) results stratified by age group.

The comparison of the Perceived Stress Scale (PSS-10) results from questionnaires stratified by age groups is shown in Table 6 and Figure 3. It was observed that the perceived helplessness was significantly higher among older patients with malignant melanoma (2.97 vs. 2.71, *p*-value = 0.036). However, perceived self-efficacy did not significantly differ between the younger and older participants, and nor did the total score.

### 3.4. Correlation Analysis

The Spearman’s rank correlation coefficients “r” by age groups, presented in Table 7, were determined for the dependent variable that evaluated the presence of depressive symptoms and anhedonia in patients with malignant melanoma. It was observed that in the elderly patients from Group B, the number of comorbidities was significantly and positively associated with the presence of depressive symptoms and anhedonia (rho = 0.291). Lower scores on the physical and mental SF-12 questionnaire determined a higher presence of depressive symptoms (rho = −0.352 and −0.273, respectively). Higher scores on the DLQI sexual difficulties and treatment difficulties also correlated significantly with the presence of depressive symptoms and anhedonia (rho = 0.341 and 0.264, respectively). Lastly, perceived helplessness on the PSS-10 questionnaire was directly associated with depressive symptoms (rho = 0.228).

## 4. Discussion

### 4.1. Literature Findings Associated with This Study

Among the main contributions of the current study is the identification of several discrepancies between younger adults and elderly patients with malignant melanoma from Romania regarding their awareness of the prevention and treatment of this aggressive skin cancer. First of all, elderly patients over 65 years old are using television as their main source of information, and they are less likely to prefer the internet or the use of smart devices to access information. The same category of patients is also less likely to visit a doctor concerning skin moles or to use sunscreen. Moreover, 80.3% of these patients from Group B believe their melanoma could have been prevented if they had a better awareness of it, indicating that some actions can be taken to address this category of exposed patients. On the other hand, the younger patients are better informed, with only 66.7% of them reporting the same thing (*p*-value = 0.031), although the effect size of the difference was small (0.139). However, their stress levels and depressive symptoms are significantly higher compared to the elderly patients, who reported more physical problems and perceived helplessness. Even though elderly patients showed statistically significant better coping methods than younger adult patients in Group A, the effect size was small.

Other studies investigated the way information reaches patients with malignant melanoma and how technological development can help them, through the use of smart devices. A recent study from Germany found that patients with malignant melanoma were mostly unaware of the existence and usefulness of health-related applications for their smart devices [12]. In the growing age of electronic health and digitalization, these findings contrast markedly with the current impression of health-related applications across medical practitioners [35]. Patients younger than 61 years old and males were substantially more likely to be engaged in obtaining knowledge and say they would download an app advised by a physician [34,36]. Individuals with a higher educational background were more likely to utilize an app than those with a lower or moderate level of education. As detailed in our study, previous studies have shown that the preponderance of patients, especially those older than 61 years, are still suspicious about the use of skin cancer applications and smart devices for searching and receiving information [37]. This mistrust might be due to a lack of suitable gadgets and a widespread fear of technological challenges, especially among the elderly.

We excluded from this study the patients with stage IV metastatic melanoma due to significantly increased levels of stress, as described in a recent study [15]. The authors detected more than fifty percent of anxious patients prior to treatment initiation. For melanoma patients who believe they need psycho-oncological assistance, it is likely that they seem to exhibit a psychometrically verified requirement, as evaluated by the Hornheide Screening Instrument.

A personal requirement for psychological assistance is of utmost significance in this situation and should be regarded as a straightforward screening method [38]. Patients should be encouraged to assess their own psycho-oncological assistance requirements. However, this question should not be substituted for the screenings since they include other questions that encourage self-reflection. Other individuals may not have recognized that they are unwell, or they may have discovered that they have no one to discuss their tough condition with [39]. The screenings may aid in provoking more self-reflection. Compliance to medication is an essential health habit for chronic patients and those undergoing chemotherapy. The social-health psychology approach and contemporary psycho-oncology trends demonstrate the impact of self on disease control and treatment adherence [21]. It has been shown that social support is essential for disease adjustment by reducing distress, depression, and the likelihood of relapse. The findings indicate that self-efficacy has a strong direct influence, as well as an indirect effect through social support and satisfaction with assistance, on patient adherence, particularly in terms of nutrition and physical activity [40].

Consistent with data from other malignancies, around one-third of malignant melanoma patients have experienced statistically considerable levels of discomfort. The level of anxiety was greatest at the time of diagnosis and soon after therapy, and it gradually reduced with time [41]. While higher levels of quality-of-life deterioration are linked with poor recovery, a rise in morbidity, or disease progression, very few authors have examined the quality of life in malignant melanoma individuals [42]. The evaluated investigations mostly concentrated on patients from specialized melanoma or other skin cancer centers that went under treatment over a short amount of time. It is not clearly researched how melanoma affects members of the general public who have survived this skin cancer for many years [43].

Even though SF-36 is the most extensively used general quality-of-life evaluation measure based on the HRQL, its psychometric qualities have not been evaluated in melanoma patients [44]; therefore, we chose to employ the abbreviated SF-12 in our research. As statistics exist for several regions, a generic tool enables comparisons between melanoma patients, other illnesses and malignancies, and the general population. As standard tools may not be sensitive enough to identify effects associated with particular disorders, the integration of a generic and specialized HRQOL instrument is the most useful.

It has been demonstrated that elderly patients with malignant melanoma exhibit more advanced Breslow index depth [45]. In the most recent period of the COVID-19 pandemic, the median age of patients with malignant melanoma was 77 years during the pre-pandemic period, compared to 53 years throughout 2020 and 2021 [46]. This discrepancy is likely attributable to the older patients’ unwillingness to attend hospital visits owing to the increased severity of SARS-CoV-2 infections in this demographic group. Other reasons for late detection of this cancer has been linked to thicker melanoma with a negative prognosis in older patients. Different variables probably contribute to the late detection of skin cancers in the elderly patient; for example, new or modifying benign skin lesions may be mistaken for melanoma for a variety of reasons, such as the invisibility of the anatomical locations of new pigmented lesions, the loss of a partner’s feedback during the home assessment, poor eyesight, a lesser emphasis on changing lesions, and confusion with benign pigmented lesions [47]. In addition, melanoma early detection initiatives have often targeted younger age groups. Therefore, investigations have shown disproportionately low rates of melanoma screening among older males, despite the fact that this age group accounts for almost half of all diagnosed cases.

### 4.2. Study Limitations and Future Perspectives

Although the current study followed strict guidelines and reported results according to validated questionnaires, there are several limitations that have to be mentioned. First, even though the number of patients with skin cancer is growing, malignant melanoma is still a rare condition in the general population. Therefore, the sample size was relatively small, decreasing the statistical power of the study and increasing the risk of type 2 errors. Further, the cross-sectional design of the study is limited to a certain moment in time when the patients are filling out the surveys, so there was no follow-up or serial measurement using questionnaires. Another limitation of the study is the imbalance between the number of participants in the two study groups, due to many elderly patients refusing to participate, or not fitting the inclusion criteria. Overall, throughout the study, the effect size of the difference between the two study groups was insufficient; therefore, the practical significance of these findings is limited.

Despite the aforementioned limitations, the current study brings important insights into the coping strategies, quality of life, and stress levels between younger adults and elderly patients with malignant melanoma. The study also managed to evaluate the level of information, misinformation, and disease awareness that exists among younger adults and elderly patients with malignant melanoma. The information presented here can be expanded for other populations of patients with different types of malignancies. Moreover, these findings can be helpful for health promotion among adults and elderly patients, based on the way they receive information and the way their cognition identifies stressors and other dimensions of quality of life [48].

## 5. Conclusions

Regarding the increasing global elderly population, new initiatives and choices are necessary. The older population should be a primary focus of awareness efforts for malignant melanoma, and television should serve as the primary channel of these awareness programs to encourage a wider use of sunscreen, avoidance of too much sun exposure, and paying more physician visits to check for malignant moles. On the other hand, patients younger than 65 who are more prone to encounter psychological issues after receiving the diagnosis of melanoma are a group that is more educated and more aware of the diagnosis and who should be given specific psycho-oncological assistance.

## Figures and Tables

**Table 1:** Comparison of demographical characteristics among patients with malignant melanoma.

Variables *	Group A (*n* = 162)	Group B (*n* = 76)	Significance
Demographics			
Gender (women)	74 (45.7%)	36 (47.4%)	0.807
Age, mean (mean ± SD) **	43.0 ± 13.9	67.2 ± 7.1	<0.001
Area of residence (urban)	91 (56.2%)	53 (69.7%)	0.045
Relationship status (married)	138 (85.2%)	71 (93.4%)	0.070
Substance use			
Frequent alcohol consumption	20 (12.3%)	17 (22.4%)	0.046
Smoking	53 (32.7%)	29 (38.2%)	0.410
Comorbidities			<0.001
0–1	116 (71.6%)	18 (23.7%)	
2	21 (13.0%)	31 (40.8%)	
≥3	25 (15.4%)	27 (35.5%)	
History of major depression	19 (11.7%)	6 (7.9%)	0.368
Depressed mood or anhedonia	71 (43.8%)	49 (28.9%)	0.028
Cancer staging			0.150
0 (In situ)	5 (3.1%)	4 (5.3%)	
I	13 (8.0%)	5 (6.6%)	
II	41 (25.3%)	29 (38.2%)	
III	103 (63.6%)	38 (50.0%)	

* Data compared with the Chi-square or Fisher’s exact test unless specified differently; ** Data analyzed with Student’s *t*-test; SD—Standard Deviation.

**Table 2:** Analysis of information, misinformation, and disease awareness as viewed by younger adults and elderly patients with malignant melanoma.

Questions (Answer—Yes)	Group A (*n* = 162)	Group B (*n* = 76)	(V) Significance
Do you know someone with malignant melanoma?	8 (4.9%)	2 (2.6%)	(0.054) 0.408
Did you know about malignant melanoma before diagnosis?	64 (39.5%)	22 (28.9%)	(0.102) 0.113
Do you think melanoma is contagious?	19 (11.7%)	5 (6.6%)	(0.079) 0.219
Do you believe melanoma can be cured?	79 (48.8%)	31 (40.8%)	(0.181) 0.249
Is malignant melanoma caused by sun exposure?	85 (52.2%)	26 (34.2%)	(0.170) 0.008
Do you use a smartphone, tablet, or personal computer?	147 (90.7%)	52 (68.4%)	(0.281) < 0.001
Do you prefer television as source of information?	53 (32.7%)	69 (90.8%)	(0.541) < 0.001
Do you prefer the internet as source of information?	120 (74.1%)	37 (48.7%)	(0.249) < 0.001
Do you use sunscreen?	42 (25.9%)	11 (14.5%)	(0.128) 0.047
Did you ever visit a doctor to evaluate your skin moles?	38 (23.5%)	7 (9.2%)	(0.167) 0.009
Do you believe your melanoma could have been prevented if someone had told you how to protect?	108 (66.7%)	61 (80.3%)	(0.139) 0.031

Chi-square or Fisher’s exact test; V—Cramer’s V effect size.

**Table 3:** Comparison of COPE-60 questionnaire results by age group.

Questions (Yes)	Likelihood	Group A (*n* = 162)	Group B (*n* = 76)	(V) Significance
Engagement				(0.093) 0.147
	Low (1–2)	93 (57.4%)	36 (47.4%)	
	High (3–4)	69 (42.6%)	40 (52.6%)	
Disengagement				(0.135) 0.036
	Low (1–2)	64 (39.5%)	41 (53.9%)	
	High (3–4)	98 (60.5%)	35 (46.1%)	
Emotion-focused				(0.075) 0.241
	Low (1–2)	70 (43.2%)	39 (51.3%)	
	High (3–4)	92 (56.8%)	37 (48.7%)	
Problem-focused				(0.153) 0.018
	Low (1–2)	73 (45.1%)	22 (28.9%)	
	High (3–4)	89 (54.9%)	54 (71.1%)	

Data were compared using the Chi-square or Fisher’s exact test; V—Cramer’s V effect size.

**Table 4:** Comparison of quality of life physical and mental scores based on the SF-12 questionnaire stratified by age group.

Physical and Mental Health (Mean ± SD)	Group A (*n* = 162)	Group B (*n* = 76)	t Statistic, df, SE	Significance (95% CI), *p*-Value
Physical Score	55.3 ± 7.6	50.8 ± 8.0	t = 4.18, df = 236, SE = 1.07	(2.83, 6.61) <0.001
Mental Score	54.1 ± 8.5	56.6 ± 6.9	t = 2.24, df = 236, SE = 1.11	(−4.69, −0.32) 0.026
Total Score	54.7 ± 8.1	53.7 ± 7.5	t = 0.90, df = 236, SE = 1.10	(−1.16, 3.16) 0.364

Data compared with Student’s t-test; SD—Standard Deviation; SE—Standard Error of the mean difference; df—Degrees of freedom; CI—Confidence Interval.

**Table 5:** Comparison of Dermatology Life Quality Index (DLQI) questionnaire results stratified by age group.

Answers (a Lot & Very Much)	Group A (*n* = 162)	Group B (*n* = 76)	(V) Significance
Item 1 (sore, itchy, painful)	22 (13.6%)	9 (11.8%)	(0.024) 0.710
Item 2 (embarrassment)	26 (16.0%)	5 (6.6%)	(0.131) 0.043
Item 3 (shopping/home)	3 (1.9%)	5 (6.6%)	(0.122) 0.059
Item 4 (clothes)	19 (11.7%)	5 (6.6%)	(0.064) 0.218
Item 5 (social activities)	38 (23.5%)	11 (14.5%)	(0.103) 0.110
Item 6 (sport)	12 (7.4%)	9 (11.8%)	(0.072) 0.260
Item 7 (working/studying)	25 (15.4%)	8 (10.5%)	(0.066) 0.307
Item 8 (interpersonal problems)	34 (21.0%)	17 (22.4%)	(0.0159) 0.808
Item 9 (sexual difficulties)	41 (25.3%)	35 (46.1%)	(0.207) 0.002
Item 10 (treatment difficulties)	48 (29.6%)	51 (67.1%)	(0.354) < 0.001

Data compared with Chi-square or Fisher’s exact test; The test compares the proportion of patients who answered with “a lot” or “very much” on the DLQI questions; V—Cramer’s V effect size.

**Table 6:** Comparison of Perceived Stress Scale (PSS-10) questionnaire results stratified by age group.

Factors (Mean ± SD)	Group A (*n* = 162)	Group B (*n* = 76)	t Statistic, df, SE	Significance (95% CI), *p*-Value
Factor 1 (Perceived Helplessness)	2.71 ± 0.83	2.97 ± 0.92	t = 2.17, df = 236, SE = 0.13	(−0.49,−0.02) 0.036
Factor 2 (Perceived Self-Efficacy)	2.94 ± 0.89	2.81 ± 0.84	t = 1.06, df = 236, SE = 0.12	(−0.10,0.36) 0.286
Total	2.82 ± 0.86	2.89 ± 0.88	t = 0.58, df = 236, SE = 1.12	(−0.30,0.16) 0.561

Data compared with Student’s t-test; SD—standard deviation; SE—Standard Error of the mean difference; df—Degrees of freedom; CI—Confidence Interval. Perceived Helplessness comprises negative questions (1, 2, 3, 6, 9, and 10); Perceived Self-Efficacy comprises positive questions (4, 5, 7, and 8).

**Table 7:** Correlation analysis by age groups.

Factors	Group A (Depressive Symptoms and Anhedonia)	Group B (Depressive Symptoms and Anhedonia)
Area of residence (rural)	0.081	0.164
Frequent alcohol consumption	0.094	0.106
Number of comorbidities	0.078	(ES = 0.084) 0.291 *
COPE-60—Disengagement coping methods	(ES = 0.112) 0.336 *	0.184
COPE-60—Problem-focused coping methods	0.137	0.120
SF-12 (physical score)	0.191	(ES = 0.123) −0.352 *
SF-12 (mental score)	(ES = 0.198) −0.446 *	(ES = 0.074) −0.273 *
DLQI—Item 2 (embarrassment)	0.168	0.039
DLQI—Item 9 (sexual difficulties)	(ES = 0.060) 0.245 *	(ES = 0.116) 0.341 *
DLQI—Item 10 (treatment difficulties)	0.130	(ES = 0.069) 0.264 *
PSS-10—Perceived Helplessness	0.059	(ES = 0.051) 0.228 *

* Statistically significant Spearman’s Rank correlation coefficient rho (*p*-value < 0.05); ES—Effect Size.

## Data Availability

Data available on request from the corresponding author.
